# Clinical Course of Iris Retraction Syndrome Associated With Rhegmatogenous Retinal Detachment: A Report of Three Cases

**DOI:** 10.7759/cureus.91977

**Published:** 2025-09-10

**Authors:** Keigo Takagi, Kazunobu Sugihara, Mizuki Iida, Kana Murakami, Chisako Ida, Hinako Ohtani, Masaki Tanito

**Affiliations:** 1 Department of Ophthalmology, Shimane University Faculty of Medicine, Izumo, JPN

**Keywords:** anterior segment optical coherence tomography, ciliary body detachment, hypotony, intraocular pressure, iris retraction syndrome, proliferative vitreoretinopathy, rhegmatogenous retinal detachment, uveitis-like presentation

## Abstract

This report describes three cases of iris retraction syndrome (IRS) associated with long-standing rhegmatogenous retinal detachment (RRD), all presenting with hypotony, abnormally deep anterior chambers, and ciliary body detachment, often accompanied by dense cataracts and uveitis-like findings that initially obscured the underlying pathology. In each case, anti-inflammatory therapy led to partial resolution of anterior segment abnormalities, subsequently revealing RRD that required pars plana vitrectomy with or without combined cataract surgery. Postoperatively, most cases maintained stable IOP, although one developed secondary ocular hypertension necessitating glaucoma therapy. These cases highlight that IRS can mask its primary cause; careful evaluation for RRD or other causes is warranted when posterior iris bowing is observed in hypotonic eyes, and long-term IOP monitoring remains important even after successful treatment of the underlying pathology.

## Introduction

Iris retraction syndrome (IRS) is a rare but clinically significant condition characterized by posterior bowing of the iris and an abnormally deep anterior chamber (AC). Campbell first described this phenomenon in 1984 in a patient with rhegmatogenous retinal detachment (RRD) [1]. Since then, IRS has been reported not only in association with RRD, but also in inflammatory ocular diseases and following intraocular surgery [2-6]. The proposed pathogenesis involves abnormal aqueous humor (AH) dynamics; when AH exits the eye more rapidly than it is produced, a negative pressure gradient may develop behind the iris, resulting in its retraction.

Although more than 40 years have passed since the initial description, IRS remains an under-recognized condition due to its rarity and diverse underlying causes. Diagnosis and management can therefore be challenging, particularly at the initial presentation. Case reports describing the clinical course of IRS are valuable for improving recognition and guiding management strategies.

Here, we present three cases of IRS associated with long-standing RRD, detailing their clinical features, management approaches, and outcomes.

## Case presentation

Case 1

A 52-year-old Japanese male presented to a local ophthalmologist with a chief complaint of decreased visual acuity in his right eye (RE). He had first noticed the reduction in vision two years earlier, which had gradually worsened over the past six months. At the local clinic, he was diagnosed with a severe cataract in the RE and was referred to our hospital for further evaluation and treatment.

**Figure 1:**
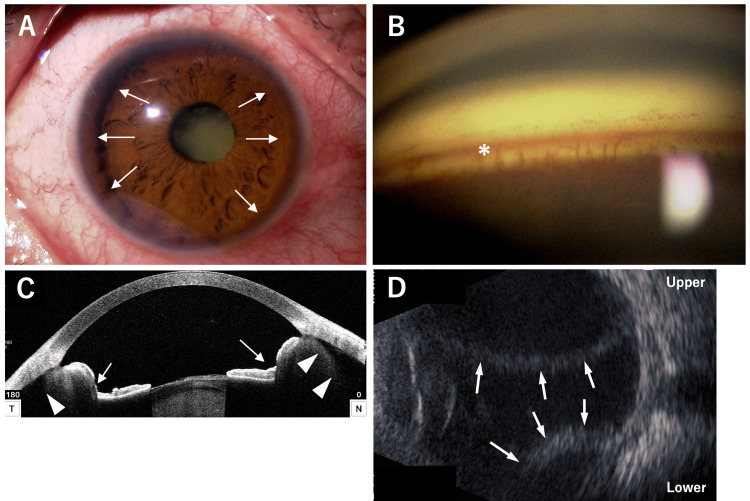
Slit-lamp photograph (A), gonioscopy (B), AS-OCT (C), and B-scan ultrasonography (D) of the right eye at the initial visit. (A) Anterior segment photograph demonstrates conjunctival hyperemia, an abnormally deep anterior chamber, a mature cataract, and circumferential iris retraction (arrows). (B) Gonioscopy demonstrates hyperemia of the trabecular meshwork (*). (C) Anterior segment optical coherence tomography (AS-OCT) shows posterior bowing of the iris (arrows) and circumferential ciliary body detachment (arrowheads). T, temporal side; N, nasal side. (D) B-scan ultrasonography reveals choroidal detachment (arrows); no obvious retinal detachment is observed.

At the initial visit to our hospital, his best-corrected visual acuity (BCVA) was hand motion in the RE and 1.2 in the left eye (LE). Intraocular pressure (IOP) was 8 mmHg in the RE and 15 mmHg in the LE. Slit-lamp examination of the RE revealed a markedly deeper AC compared to the LE. The iris showed kinking and was adherent to the anterior surface of the lens, with posterior synechiae involving approximately 270 degrees of the iris circumference, sparing only the nasal side. Conjunctival hyperemia, ciliary injection, and a mature cataract were also noted (Figure 1A). In the LE, no conjunctival hyperemia was observed, and the lens was clear. Gonioscopy of the RE revealed an abnormally wide-open angle, posterior retraction of the iris, and hyperemia of the trabecular meshwork (TM) (Figure 1B). In contrast, gonioscopy of the LE demonstrated a normally wide-open angle without TM hyperemia. Anterior segment optical coherence tomography (AS-OCT; CASIA2 Advance, Tomey, Nagoya, Japan) of the RE (Figure 1C) revealed an abnormally deep AC, posteriorly retracted iris (arrows), and a 360-degree ciliary body detachment (arrowheads). The axial length (AL; OA-2000, Tomey) was 22.81 mm in the RE and 23.31 mm in the LE. The corneal endothelial cell density (CECD; EM-3000 specular microscope, Tomey) was 722 cells/mm² in the RE and 2679 cells/mm² in the LE; the low CECD value in the RE was considered inaccurate due to hypotony. The central corneal thickness (CCT; EM-3000 specular microscope) was 598 µm in the RE and 504 µm in the LE. B-scan ultrasonography (UD-800, Tomey) of the RE (Figure 1D) showed marked choroidal detachment (arrows) and possible mild scleral thickening; no clear evidence of retinal detachment was observed at that time. Given the presence of hypotony, conjunctival hyperemia, and mild scleral thickening, ocular inflammation of unknown etiology was suspected. On the day of the initial visit, a sub-Tenon’s injection of triamcinolone acetonide (20 mg; Kenakort-A, Bristol Myers Squibb, Tokyo, Japan) was administered, followed by topical 1% atropine twice daily and topical 0.1% betamethasone sodium phosphate three times daily.

**Figure 2:**
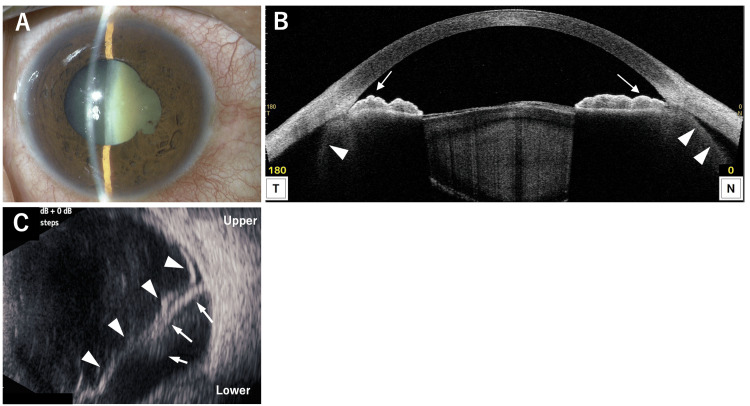
Slit-lamp photograph (A), AS-OCT (B), and B-scan ultrasonography (C) of the right eye after anti-inflammatory treatment. (A) Four days after the initial visit, conjunctival hyperemia has resolved, anterior chamber depth has normalized, and posterior synechiae are present. (B) Anterior segment optical coherence tomography (AS-OCT) at the same time point shows improvement in posterior bowing of the iris (arrows) and reduction, but persistence of ciliary body detachment (arrowheads). (C) Eleven days after the initial visit, B-scan ultrasonography shows reduced choroidal detachment (arrows) and wavy high-reflective signals indicating retinal detachment (arrowheads).

At a follow-up visit four days later, the AC depth in the RE had normalized, and both conjunctival and ciliary hyperemia had improved (Figure 2A). AS-OCT of the RE (Figure 2B) showed improvement in the posterior iris retraction (arrows) and partial resolution of the ciliary body detachment (arrowheads). The anterior chamber flare (ACF; FM-600 laser flare meter, Kowa, Nagoya, Japan) was 296.0 pc/msec in the RE and 7.7 pc/msec in the LE. At the third visit, 11 days after the initial presentation, B-scan ultrasonography (Figure 2C) demonstrated that the choroidal detachment persisted but had reduced in extent (arrows), and retinal detachment was suspected based on the presence of wavy, high-reflective signals (arrowheads). Fundus observation was still not possible due to the severe cataract. The ACF in the RE at this time was 327.3 pc/msec. IOP was 11 mmHg in the RE and 18 mmHg in the LE.

**Figure 3:**
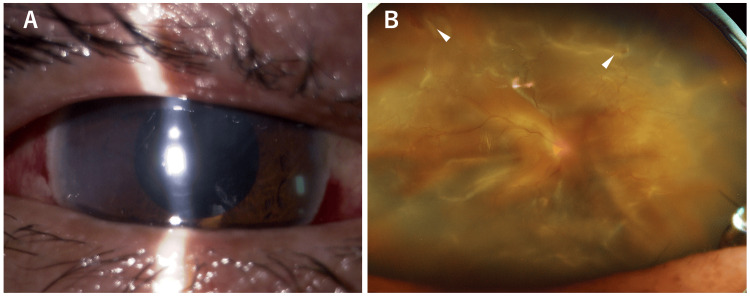
Slit-lamp photograph (A) and ultra-widefield fundus photograph (B) of the right eye after cataract surgery. (A) Anterior segment photograph shows aphakia. (B) Ultra-widefield fundus photograph reveals total retinal detachment and causative retinal breaks (arrowheads).

To enable fundus observation and facilitate resolution of the choroidal detachment, cataract surgery combined with drainage of suprachoroidal fluid was performed on the same day. During the surgery, a posterior capsule rupture occurred, necessitating anterior vitrectomy. The procedure was concluded with the eye left aphakic (Figure 3A). On the day after cataract surgery, fundus visualization became possible and revealed a rhegmatogenous retinal detachment (RRD) (Figure 3B). A 25-gauge pars plana vitrectomy (PPV) with silicone oil tamponade and sulcus-fixated intraocular lens (IOL) implantation was scheduled for 10 days later (three weeks after the initial presentation).

**Figure 4:**
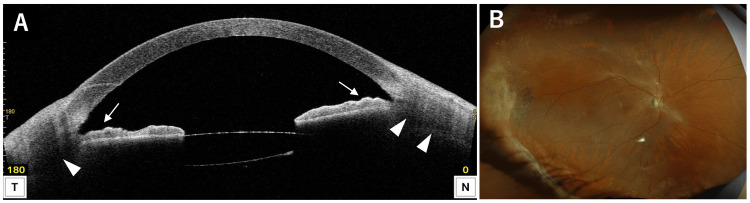
AS-OCT (A) and ultra-widefield fundus photograph (B) of the right eye after pars plana vitrectomy. (A) Anterior segment optical coherence tomography (AS-OCT) demonstrates normalization of iris configuration (arrows) and resolution of ciliary body detachment (arrowheads). (B) Ultra-widefield fundus photograph shows retinal reattachment.

The PPV revealed multiple horseshoe retinal tears extending across all quadrants. Postoperatively, AS-OCT of the RE (Figure 4A) demonstrated flattening of the iris (arrows), normalization of AC depth, and resolution of ciliary body detachment (arrowheads). Two weeks after the vitrectomy (five weeks after the initial visit), recurrence of the RRD occurred, requiring a second 25-gauge PPV with silicone oil tamponade. Silicone oil removal was performed 17 weeks after the second vitrectomy (22 weeks after the initial visit) (Figure 4B). After each surgery, topical levofloxacin and betamethasone sodium phosphate were administered four times daily to the RE for approximately one month. No rapid fluctuations in IOP were observed during the entire treatment course. At the final visit, eight months after the initial consultation, BCVA was 0.4 in the RE and 1.0 in the LE. IOP was 14 mmHg in the RE and 17 mmHg in the LE. The CECD in the RE was 1888 cells/mm², and the ACF was 59.5 pc/msec, suggesting a sustained elevation compared with the LE.

Case 2

A 63-year-old man presented to a local ophthalmologist complaining of blurred vision in the RE, which he had noticed five days earlier. A severe cataract was observed, and IOP was 4 mmHg in the RE. B-scan ultrasonography suggested either retinal detachment (RD) or choroidal detachment. Two weeks later, he was referred to our hospital for further evaluation and management.

**Figure 5:**
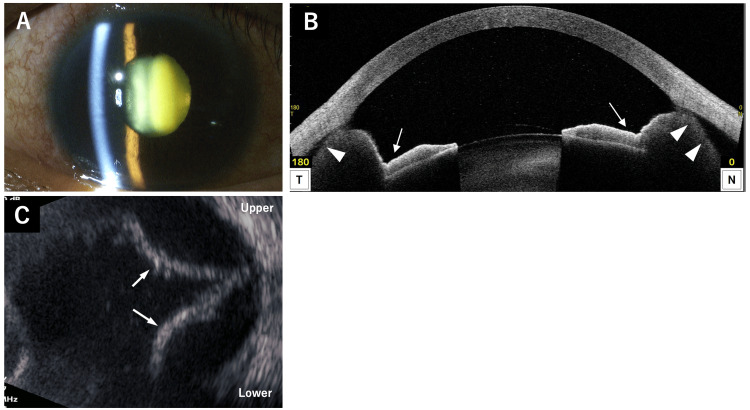
Slit-lamp photograph (A), AS-OCT (B), and B-scan ultrasonography (C) of the right eye at the initial visit. (A) Anterior segment photograph shows conjunctival hyperemia, deep anterior chamber, and a mature cataract. (B) Anterior segment optical coherence tomography (AS-OCT) demonstrates posteriorly bowed iris (arrows) and ciliary body detachment (arrowheads). (C) B-scan ultrasonography reveals choroidal detachment (arrow).

At the initial visit to our hospital, BCVA was hand motion in the RE and 0.2 in the LE. IOP was 5 mmHg in the RE and 21 mmHg in the LE. Slit-lamp examination of the RE revealed conjunctival hyperemia, a markedly deep AC compared with the LE, and a nuclear cataract (Figure 5A). The LE also had a nuclear cataract, though less advanced than that in the RE. After pupillary dilation with tropicamide and phenylephrine hydrochloride, posterior synechiae involving approximately 270° of the iris, from temporal to superior and inferonasal quadrants, were observed in the RE. AS-OCT of the RE (Figure 5B) demonstrated posterior bowing of the iris (arrows), an abnormally deep AC, and a 360° ciliary body detachment (arrowheads). AL was 26.63 mm in the RE and 27.15 mm in the LE. CECD was 1106 cells/mm² in the RE and 2616 cells/mm² in the LE. ACF was unmeasurable in the RE and 8.0 pc/msec in the LE. B-scan ultrasonography (Figure 5C) confirmed choroidal detachment (arrows), but no definitive RD was observed at that time. Based on the findings of hypotony, conjunctival hyperemia, and posterior synechiae in the RE, scleritis was suspected. Anti-inflammatory therapy was initiated with a sub-Tenon’s injection of triamcinolone acetonide (20 mg), followed by topical 1% atropine twice daily and topical 0.1% betamethasone sodium phosphate three times daily in the RE.

**Figure 6:**
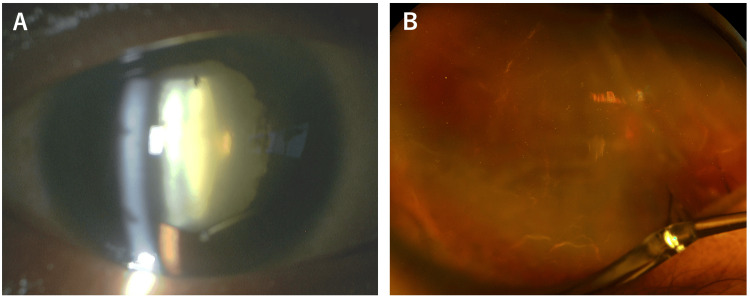
Slit-lamp photograph (A) and ultra-widefield fundus photograph (B) of the right eye after anti-inflammatory treatment. (A) Anterior segment photograph shows posterior synechiae and normalized anterior chamber depth. (B) Ultra-widefield fundus photograph reveals total retinal reattachment.

Two weeks after the initial presentation, AC depth in the RE had normalized (Figure 6A). Choroidal and ciliary body detachments had improved, but a total RRD had become apparent (Figure 6B). ACF in the RE was 365.3 pc/msec at this time. Four weeks after the initial presentation, combined cataract surgery and 25-gauge PPV with silicone oil tamponade were performed, and the retina was successfully reattached. Postoperatively, topical levofloxacin was given four times daily and topical betamethasone sodium phosphate six times daily in the RE for one month, after which the frequency of betamethasone was reduced to four times daily and continued for another month.

**Figure 7:**
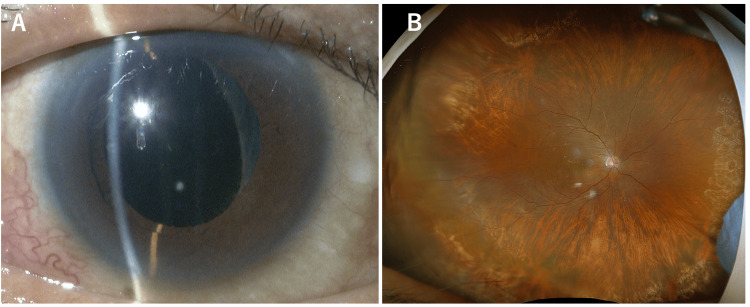
Slit-lamp photograph (A) and ultra-widefield fundus photograph (B) of the right eye after phacovitrectomy. (A) Anterior segment photograph shows normal anterior chamber depth. (B) Ultra-widefield fundus photograph shows retina successfully reattached.

Eight weeks after the initial PPV, silicone oil removal was performed using 25-gauge PPV. Postoperatively, AC depth remained normal (Figure 7A), and the retina remained attached (Figure 7B). Topical levofloxacin and betamethasone sodium phosphate were continued four times daily in the RE for one month. IOP, which had been low before surgery, returned to normal during the postoperative course. At the final follow-up, BCVA was 0.05 in the RE and 0.3 in the LE. IOP was 16 mmHg in the RE and 20 mmHg in the LE. CECD in the RE was 2571 cells/mm², and ACF was 41.1 pc/msec, indicating a sustained elevation compared with the LE.

Case 3

A 61-year-old female presented to a local ophthalmologist with a chief complaint of decreased vision in the RE over the past three to four months. A mature cataract was observed in the RE. B-scan ultrasonography revealed findings suggestive of RD. Four days after her initial presentation, she was referred to our hospital for further evaluation and treatment.

**Figure 8:**
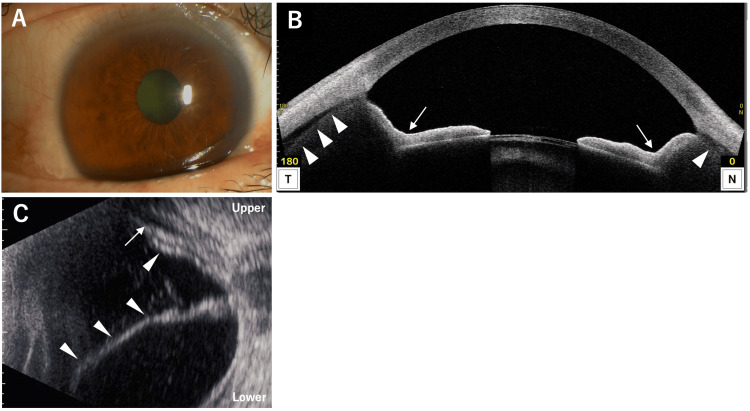
Slit-lamp photograph (A), AS-OCT (B), and B-scan ultrasonography (C) of the RE at the initial visit. (A) Anterior segment photograph shows conjunctival hyperemia and a mature cataract. (B) AS-OCT demonstrates posterior bowing of the iris (arrow) and ciliary body detachment (arrowhead). (C) B-scan ultrasonography reveals partial choroidal detachment (arrow) and obvious RD (arrowheads).

At the initial visit to our hospital, BCVA was hand motion in the RE and 0.4 in the LE. IOP was 7 mmHg in the RE and 19 mmHg in the LE. Slit-lamp examination of the RE revealed a markedly deeper AC compared with the LE and a mature cataract (Figure 8A). The LE had a nuclear cataract, and its AC depth was within normal limits. Fundus observation of the RE was not possible due to the mature cataract. AS-OCT of the RE (Figure 8B) showed characteristic posterior bowing of the iris (arrows), deepening of the AC, and a 360-degree ciliary body detachment (arrowheads). AL was 27.26 mm in the RE and 33.05 mm in the LE. CECD was 2566 cells/mm² in the RE and 2351 cells/mm² in the LE. B-scan ultrasonography of the RE (Figure 8C) showed partial choroidal detachment (arrow) and clear evidence of RD (arrowheads). Based on these findings, a diagnosis of IRS associated with RRD was made, and the patient underwent combined cataract surgery and 25-gauge PPV with silicone oil tamponade.

**Figure 9:**
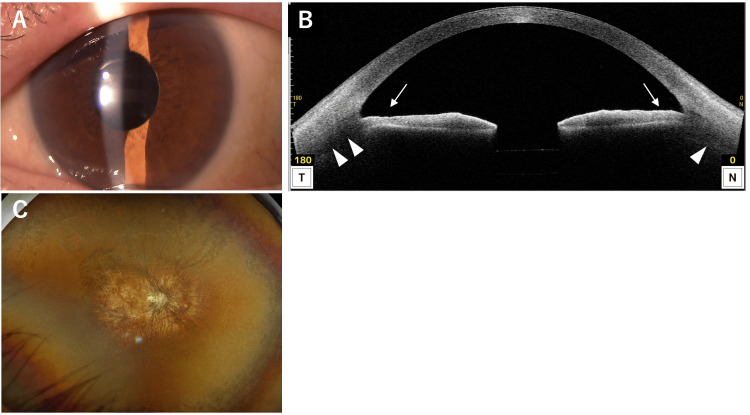
Slit-lamp photograph (A), AS-OCT (B), and ultra-widefield fundus photograph (C) of the right eye after phacovitrectomy. (A) Anterior segment photograph shows normalized anterior chamber depth. (B) Anterior segment optical coherence tomography (AS-OCT) demonstrates normalization of the iris configuration (arrows) and resolution of ciliary body detachment (arrowheads). (C) Ultra-widefield fundus photograph shows retina successfully reattached.

Intraoperatively, a paravascular retinal break was identified in the periphery of the superior vascular arcade, which was considered the cause of the RRD. The retina was successfully reattached under silicone oil tamponade. Postoperatively, slit-lamp examination showed normalization of AC depth (Figure 9A). AS-OCT (Figure 9B) demonstrated normalization of the iris configuration (arrows), restoration of AC depth, and resolution of ciliary body detachment (arrowhead). Topical levofloxacin and betamethasone sodium phosphate were administered four times daily in the RE for one month. At the follow-up visit one week after surgery, IOP in the RE had risen to 50 mmHg. Treatment was initiated with oral acetazolamide 500 mg daily and fixed-combination timolol/dorzolamide twice daily. One week later, IOP in the RE decreased to 30 mmHg, but the patient developed a skin rash on her abdomen, attributed to acetazolamide, which was then discontinued. Latanoprost once daily was added. Due to persistently elevated IOP, early silicone oil removal was scheduled. Four weeks after the first surgery (six weeks after the initial visit), silicone oil removal was performed via 25-gauge PPV. Although the retina remained reattached, IOP in the RE again rose to 30 mmHg one week after the second surgery. Fixed-combination timolol/dorzolamide was reintroduced twice daily. Six months after the initial visit, cataract surgery was performed in the LE. At the final follow-up, BCVA was 0.09 in the RE and 1.0 in the LE. IOP was 17 mmHg in the RE and 15 mmHg in the LE with ongoing fixed-combination timolol/dorzolamide in the RE. CECD was 1592 cells/mm² in the RE, and ACF was 46.5 pc/msec, indicating a sustained elevation compared with the LE.

## Discussion

IRS is a pathological condition caused by altered AH pressure gradients that result from hypotony and underlying ocular diseases [1]. From our case series, two important clinical issues can be highlighted.

First, an insufficient understanding of IRS can cause clinicians to overlook its underlying cause. This difficulty arises because IRS often presents with dense cataracts and marked choroidal detachment secondary to long-standing hypotony, which can obscure the primary pathology. When ciliary body function declines, AH production decreases [7]. AH is a complex fluid containing electrolytes, cytokines, and proteins, providing nutrients and immune components to AC tissues [8], and is also thought to help maintain lens transparency [9]. Consequently, many patients with IRS develop mature cataracts. Furthermore, for IRS to develop, an alternative outflow pathway for AH must exist. If AH outflow through such a pathway exceeds production, a reversed pressure gradient forms, resulting in the characteristic posterior bowing of the iris. These pathways often include retinal breaks or ciliary body detachment, frequently accompanied by significant choroidal detachment. In our cases, diagnosis of RRD was delayed because the choroidal detachment created a pseudo-scleral buckle effect, complicating the clinical picture. Typically, RRD presents acutely, and patients seek medical attention for visual field loss; however, in some patients, sudden visual loss may go unnoticed, which may help explain the rarity of IRS. Additionally, all our cases initially presented with uveitis-like findings, which led us to initiate anti-inflammatory therapy first in cases 1 and 2. This is likely caused by inflammatory changes from long-standing RRD, which can induce proliferative vitreoretinopathy (PVR). PVR introduces retinal pigment epithelial cells, inflammatory mediators, and growth factors onto the retinal surface, promoting contractile membrane formation [10]. The clinical usefulness of preoperative anti-inflammatory therapy for PVR is still uncertain. PVR is a key factor influencing the prognosis of IRS. Table 1 summarizes previously reported cases with documented clinical courses along with our own cases, including patient data such as visual acuity (VA) and IOP before and after treatment, underlying etiology, presence of a dense cataract, and specific glaucoma treatments. The most commonly reported causes were RRD or PVR. In reports documenting lens findings, mature cataracts were frequently observed. Pre-treatment VA, ranging from hand motion to 0.3, was generally poor, likely due to PVR and advanced cataracts.

**Table 1:** Summary of published case reports on iris retraction syndrome (IRS). This table summarizes previously published case reports on IRS and our report. * indicates that this represents a summary of nine cases, reporting the range of values from minimum to maximum. ** indicates the increase in intraocular pressure in response to anti-inflammatory treatment. ASC: anterior subcapsular cataract; CF: counting fingers; HM: hand motion; IOL: intraocular lens; IOP: intraocular pressure; LP: light perception; PVR: proliferative vitreoretinopathy; RD: retinal detachment; RRD: rhegmatogenous retinal detachment; SB: scleral buckling; SRH: subretinal hemorrhage.

Author (year)	Pre-treatment visual acuity	Pre-treatment IOP (mmHg)	Etiology	Dense cataract	Post-treatment visual acuity	Post-treatment IOP (mmHg)	Glaucoma treatment
Campbell (1984) [1]	LP	2-7*	PVR	Yes	Not improved	Not documented	No
Greenfield et al. (1995), Case 1 [2]	Not documented	18	After SB	No (IOL)	Not documented	Not documented	No
Greenfield et al. (1995), Case 2 [2]	CF	4	RRD	Yes	Not documented	Normal	No
Geyer et al. (1998), Case 1 [3]	CF	10	SRH and RD	Not documented	Not documented	18	No
Geyer et al. (1998), Case 2 [3]	CF	4	Uveitis	Not documented	Not documented	12	No
Goebel et al. (2021) [4]	0.05	6	PVR	Not documented	0.1	17	No
Petrash et al. (2022) [5]	0.3	6 to 21**	Posterior cyclitis	No (ASC)	0.7	13	Tube shunt
Kandarakis et al. (2024) [6]	0.1	3	Uveitis	No (nuclear cataract)	0.1	14	Acetazolamide
Our report, Case 1	HM	8	RRD	Yes	0.4	14	No
Our report, Case 2	HM	7	RRD	Yes	0.09	17	Yes
Our report, Case 3	HM	5	RRD	Yes	0.05	16	No

Second, prolonged hypotony can lead to atrophy of the TM-Schlemm's canal (SC) outflow pathway for AH, which may be associated with poor IOP control after resolution of the primary pathology. Johnson et al. reported that long-term hypotony after successful filtration surgery can cause narrowing of SC and impaired TM function [11]. Similarly, Ishida et al. described cases in which persistent hypotony after microhook ab interno trabeculotomy led to marked ocular hypertension after recovery of ciliary detachment, necessitating filtration surgery [12]. A similar mechanism is suspected in IRS, where long-standing ciliary body detachment suppresses AH drainage into SC, causing disuse atrophy of this outflow pathway. In Table 1, all but one case exhibited hypotony before treatment. In our series, only one of the three cases required subsequent glaucoma treatment, and in previous reports, two of eight cases required such intervention. Therefore, the frequency of postoperative IOP elevation in IRS patients may be lower than in glaucomatous eyes after prolonged hypotony, possibly because most IRS cases initially have preserved TM/SC function. However, as some IRS patients do develop postoperative IOP elevation, careful postoperative IOP monitoring is essential to prevent optic nerve damage.

## Conclusions

In conclusion, the clinical presentation of IRS can obscure the underlying cause, and in some cases, glaucoma treatment is required even after resolution of the initial pathology. When posterior iris retraction is observed in the setting of hypotony, clinicians should consider possible underlying conditions such as RRD or uveitis. A thorough understanding of the pathophysiology of IRS facilitates more direct and appropriate therapeutic decision-making.
